# Supplementary material for: Cinnamaldehyde Enhances Antimelanoma Activity through Covalently Binding ENO1 and Exhibits a Promoting Effect with Dacarbazine
Source: Cancers (Basel). 2020 Jan 29;12(2):311. doi: 10.3390/cancers12020311 (PMC7072165; doi:10.3390/cancers12020311)
Supplement: Supplementary file 1 [file cancers-12-00311-s001.pdf]

Supplementary Material

Cinnamaldehyde Enhances Antimelanoma Activity Through Covalently Binding ENO1 and Exhibits a Promoting Effect with Dacarbazine

Weiyi Zhang, Jie Gao, Chuanjing Cheng, Man Zhang[1], Wenjuan Liu, Xiaoyao Ma, Wei Lei, Erwei Hao, Xiaotao Hou, Yuanyuan Hou and Gang Bai

Description on Supplemental Results

Part One

In this section, we have provided supplementary materials and tables mentioned in the result of the manuscript. The reliability of experimental results was supported as supplementary evidence.

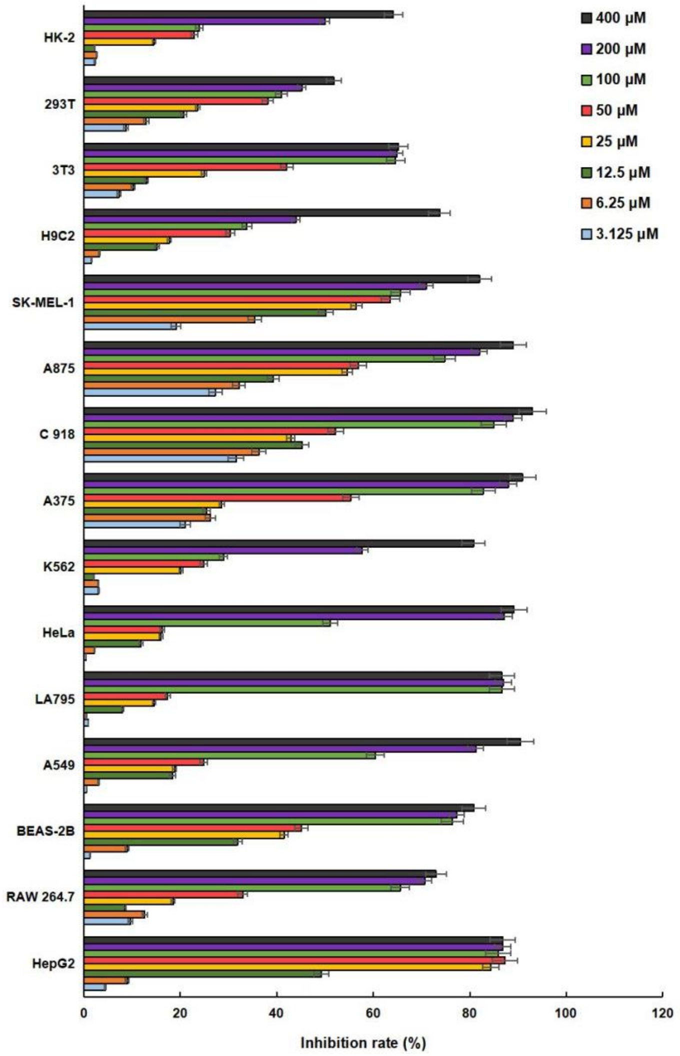

Figure S1. Cytotoxic effects of CA on a variety of tumor cells and normal cells. Cells were treated with 3.125, 6.25, 12.5, 25, 50, 100, 200 or 400 μM CA for 72 h, and the inhibition rate of CA was measured.

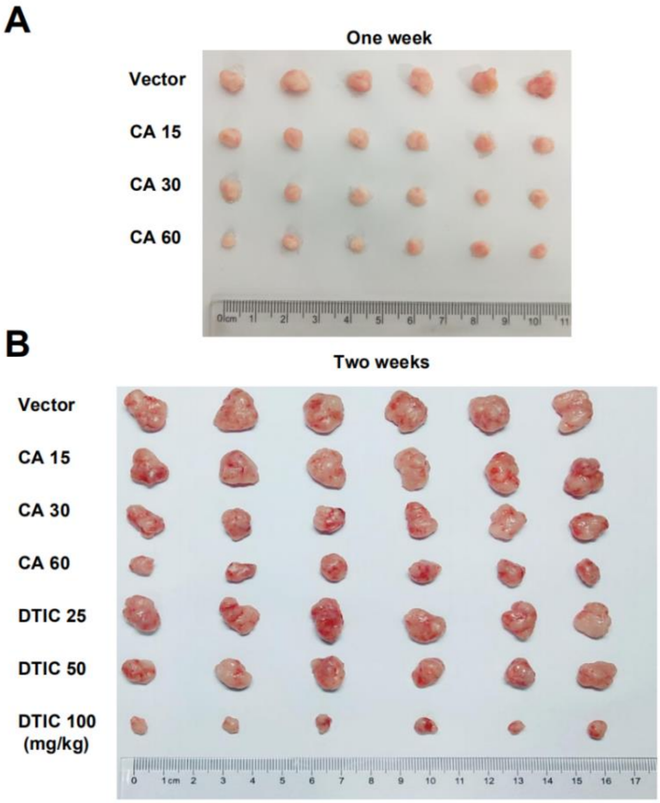

Figure S2. DTIC and CA inhibited the growth of A375 tumor xenografts. (**A**) In the preliminary test, A375 cells were subcutaneously injected into the upper left flank region of nude mice. When the tumors had formed for one week, CA (15, 30 or 60 mg/kg/day) was treated for one week. (**B**) A375 cells were subcutaneously injected into the upper left flank region of nude mice. When the tumors had formed for one week, mice were treated with DTIC (25, 50 or 100 mg/kg/day) and CA (15, 30 or 60 mg/kg/day) for two weeks. PBS was used as a negative control. Finally, mice were sacrificed by decollement, and tumor tissues were stripped. (n = 6, expressed as means ± standard deviation).

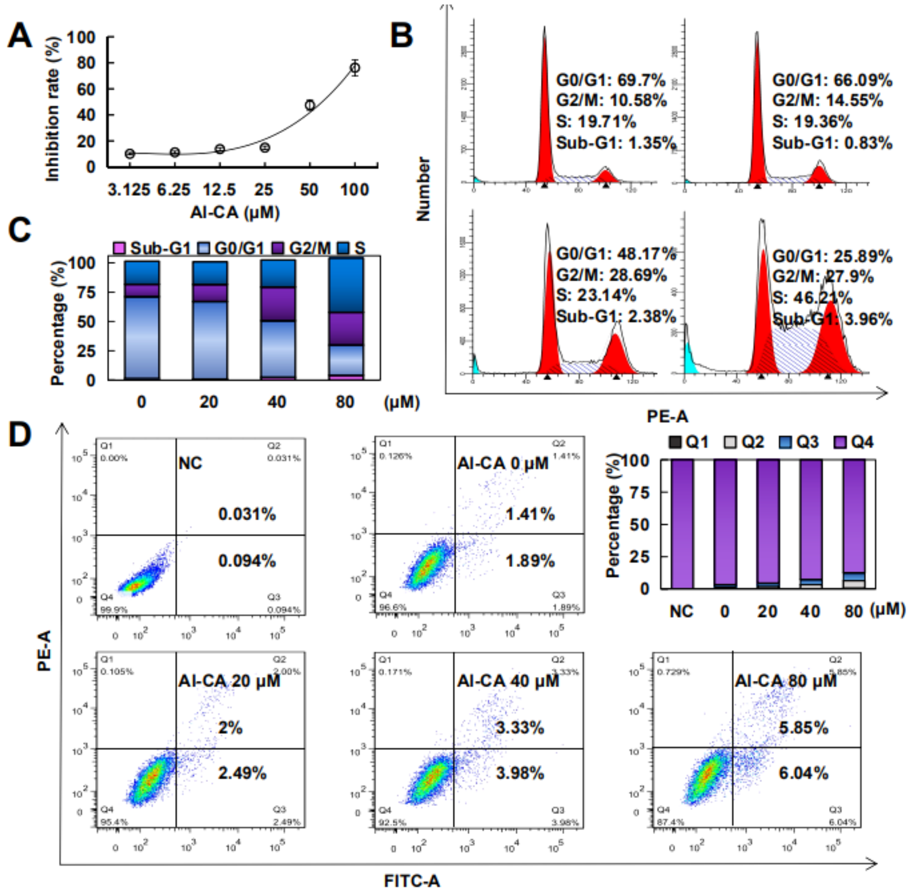

Figure S3. Al-CA arrests the cell cycle in A375 cells and stimulates apoptosis. (A) The cytotoxicity of Al-CA was evaluated against the A375 cell line. Cells were treated Al-CA for 72 h, and then the cell viability was assayed. (B) Al-CA induces G2/M and S phase arrest in A375 cells. Cells were treated without or with 0, 20, 40, or 80 μM Al-CA for 24 h. The DNA contents of the cells were determined with a flow cytometry system. (C) The histograms show the percentage of cells in Sub-G1, G0/G1, G2/M, and S phase after treatment with Al-CA. (D) Flow cytometric analysis of Al-CA-induced apoptosis in A375 cells by double-staining with Annexin V-FITC/PI. Cells were untreated or treated with 0, 20, 40, or 80 μM Al-CA for 24 h. Histograms show the percentage of different regions, “Q2” and “Q3” represent late and early apoptosis, respectively.

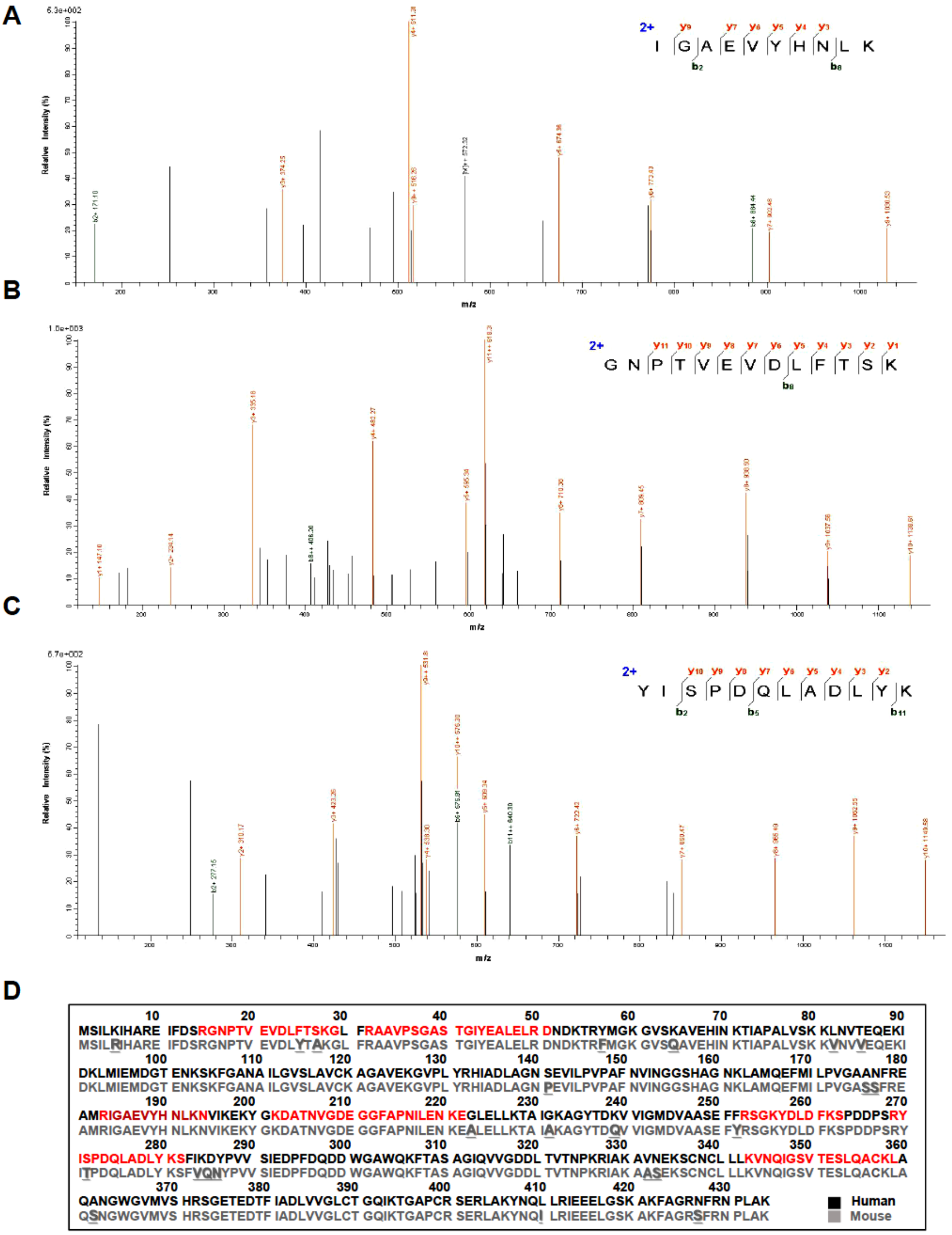

Figure S4. (A/B/C) The captured protein target from Figure 2B was recovered from the gel and sent for HPLC-mass spectrometry identification. The main peptide fragments identified by protein mass spectrometry were listed. (D) Sequence alignment analysis for ENO1 protein in human and mouse. The underlined portion is the differential amino acid. The red color represents the sequence fragment that was identified by protein mass spectrometry.

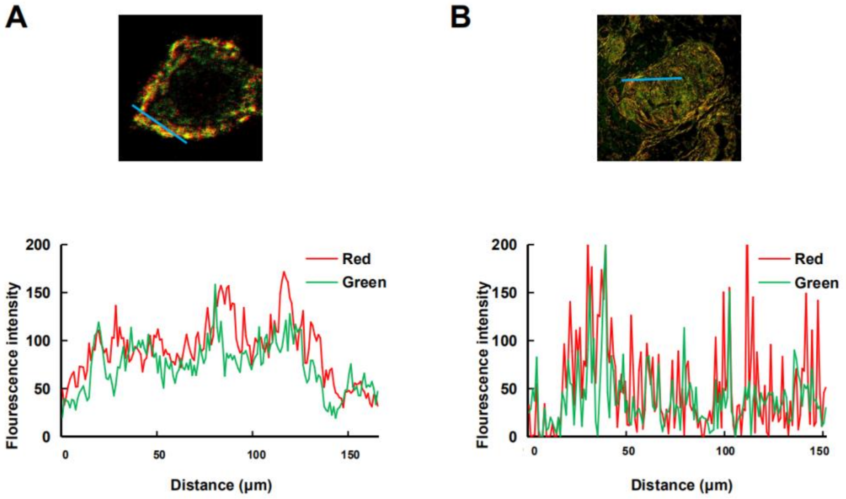

Figure S5. CA colocalized with ENO1 both in vitro (A) and in vivo (B). The pseudo red color represents ENO1, which was stained by Alexa Fluor 594, and the Al-CA probe took on a pseudo green color via the click reaction. The statistical result of fluorescence sensitivity was used Image J software to analyze and it was showed a high overlapping (>90%) at the underline position (blue), which was in Figure 3C/D.

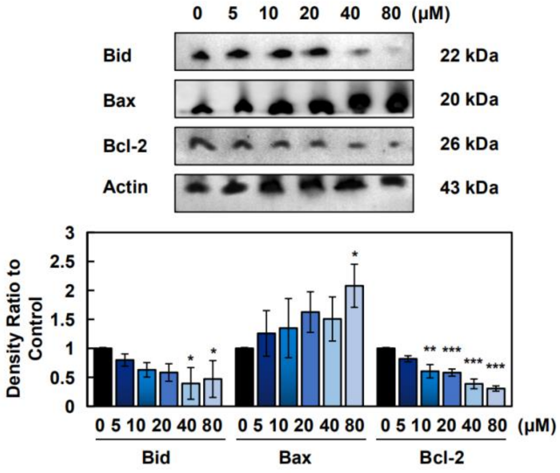

Figure S6. CA induced apoptosis. A375 cells were treated with the indicated concentrations of CA for 24 h, and the apoptosis-related proteins, Bid, Bax and Bcl-2 were detected by Western blotting. Histograms show the intensity of apoptosis-related protein bands (n = 3). * $p < 0.05$, ** $p < 0.01$, *** $p < 0.001$ versus “0” group.

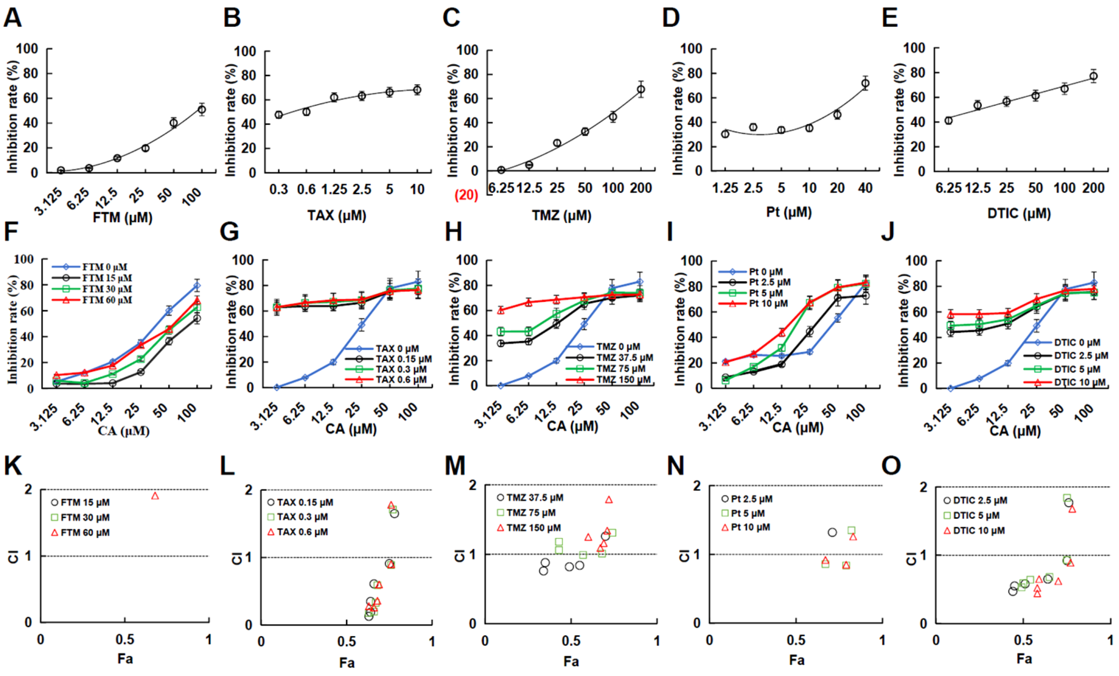

Figure S7. CA showed a synergistic effect with some chemotherapeutic medicines. (A/B/C/D/E) A375 cells were treated with FTM, TAX, TMZ, Pt, or DTIC for 72 h, respectively, and the inhibition rate was measured (n = 3). (F/G/H/I/J) A375 cells were treated with FTM, TAX, TMZ, Pt and DTIC, respectively, in the presence of CA (3.125, 6.25, 12.5, 25, 50, or 100 μM) for 72 h, and the inhibition of CA was measured (n = 3). (K/L/M/N/O) The cooperativity index (CI) value reflecting the synergism of two medicines was calculated using CompuSyn software. CI values of <1, 1, and >1 indicate synergistic, additive, and antagonistic effects, respectively.

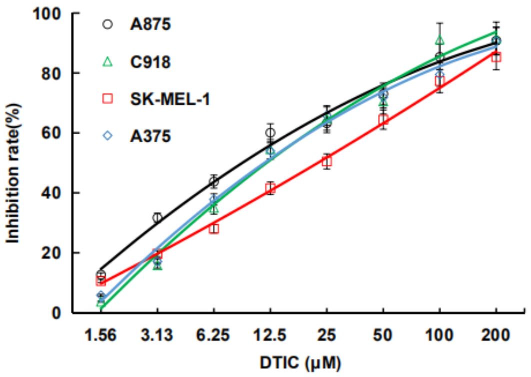

Figure S8. DTIC shows similar cytotoxicity against different kinds of melanoma cell lines. The selected cell lines were treated with different concentrations of CA for 72 h, and the cell viability was assayed by the CCK-8 method.

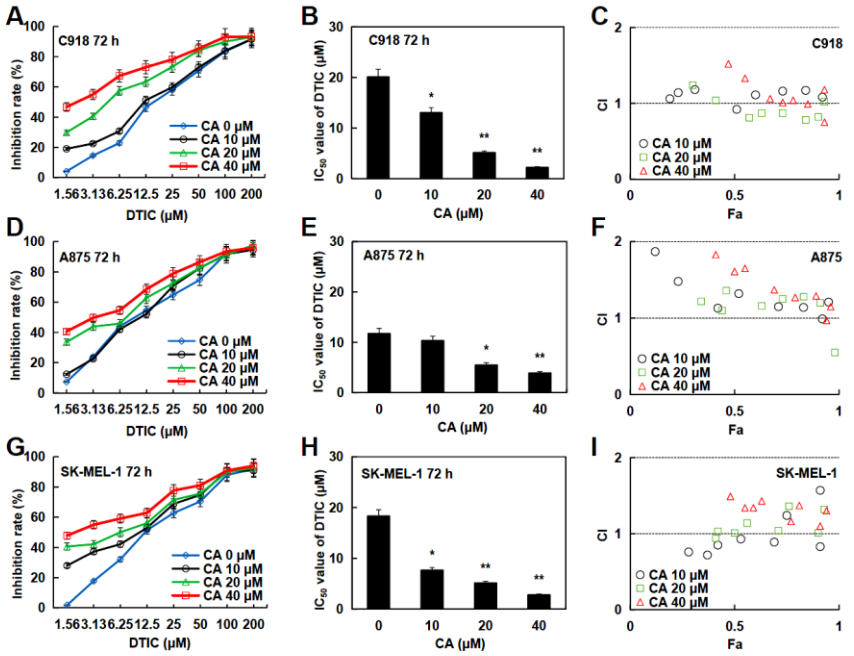

Figure S9. CA showed a synergistic effect with DTIC. (A/B, D/E, G/H) A875, C918 and SK-MEL-1 cells were treated with DTIC for 72 h, respectively, after which the inhibition rate was assayed and the IC50 value was calculated (n = 3). (C, F, I) The CI values reflecting the synergism of the two medicines were calculated using CompuSyn software. CI values of <1, 1, and >1 indicate synergistic, additive, and antagonistic effects, respectively.

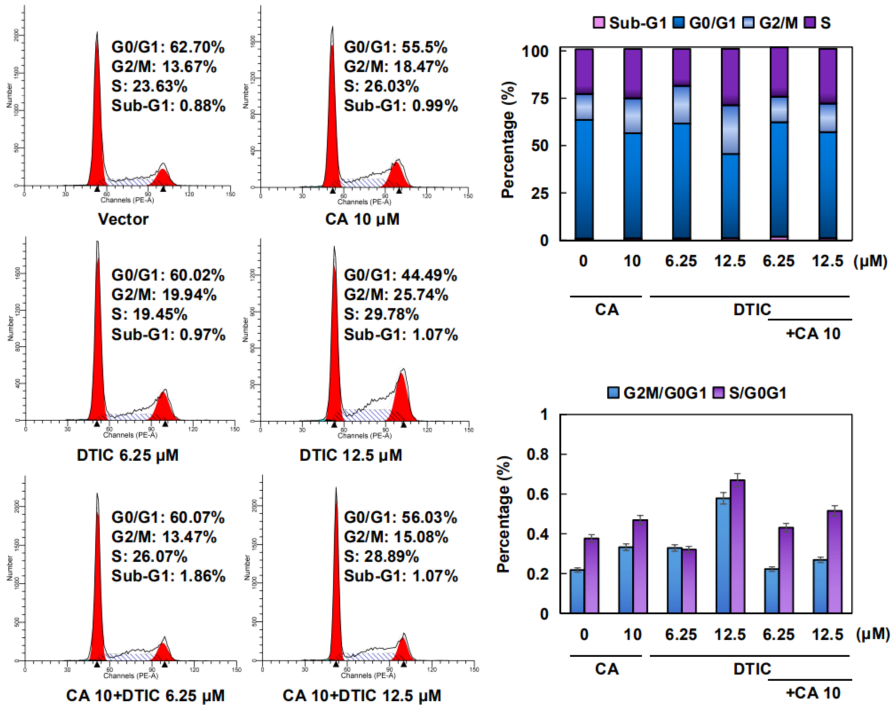

Figure S10. Cells were treated with 6.25 or 12.5 μM DTIC and with 0 or 10 μM CA for 24 h and were analyzed with a flow cytometry system, and the G2M or S phase ratio to G0/G1 was calculated, respectively.

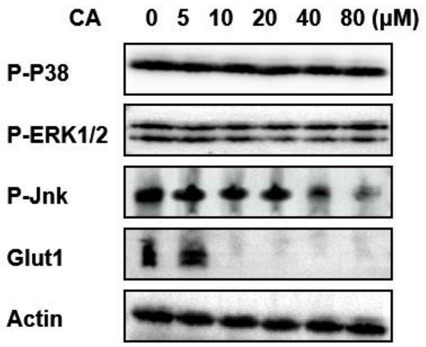

Figure S11. A375 cells were treated with 0, 5, 10, 20, 40 or 80 μM CA for 24 h, and the related protein were detected by Western blotting. Results showed that after CA treatment, the phosphorylation (P-) of JNK was decreased at high concentration (>40 μM), and the change of P-ERK1/2 and P-P38 were not evident in the downstream of Pi3K/Akt pathway. The expression of Glut1 was observably suppressed by CA (>10 μM).

Supplement Table 1. The CI value of CA with different chemotherapeutic drugs.

CA (µM)	FTM (15 µM)		FTM (30 µM)		FTM (60 µM)	
	Fa	CI	Fa	CI	Fa	CI
3.125	0.04	3.92	0.06	4.64	0.1	5.28
6.25	0.04	5.12	0.04	7.85	0.12	4.89
12.5	0.04	7.52	0.11	4.13	0.18	3.89
25	0.13	4.28	0.23	2.93	0.33	2.60
50	0.36	2.56	0.45	2.10	0.46	2.45
100	0.54	2.63	0.63	2.06	0.68	1.91
	DTIC (2.5 µM)		DTIC (5 µM)		DTIC (10 µM)	
	Fa	CI	Fa	CI	Fa	CI
3.125	0.44	0.47	0.49	0.53	0.58	0.44
6.25	0.45	0.55	0.50	0.59	0.58	0.52
12.5	0.51	0.58	0.54	0.64	0.59	0.65
25	0.64	0.65	0.65	0.68	0.70	0.62
50	0.75	0.92	0.75	0.93	0.77	0.89
100	0.76	1.77	0.75	1.84	0.78	1.68
	TAX (0.15 µM)		TAX (0.3 µM)		TAX (0.6 µM)	
	Fa	CI	Fa	CI	Fa	CI
3.125	0.63	0.13	0.63	0.18	0.63	0.28
6.25	0.64	0.19	0.66	0.20	0.66	0.26
12.5	0.64	0.35	0.67	0.33	0.68	0.36
25	0.66	0.61	0.68	0.59	0.69	0.60
50	0.75	0.91	0.76	0.89	0.76	0.90
100	0.78	1.65	0.77	1.71	0.76	1.78
	TMZ (37.5 µM)		TMZ (75 µM)		TMZ (150 µM)	
	Fa	CI	Fa	CI	Fa	CI
3.125	0.34	0.76	0.43	1.06	0.60	1.25
6.25	0.35	0.88	0.43	1.18	0.67	1.09
12.5	0.49	0.82	0.57	0.99	0.69	1.16
25	0.55	0.84	0.68	1.01	0.71	1.34
50	0.70	1.26	0.74	1.31	0.72	1.79
100	0.72	2.24	0.74	2.24	0.73	2.70
	Pt (2.5 µM)		Pt (5 µM)		Pt (10 µM)	
	Fa	CI	Fa	CI	Fa	CI
3.125	0.09	42.11	0.06	227.41	0.2	18.57
6.25	0.13	18.12	0.17	16.43	0.27	8.20
12.5	0.19	9.09	0.32	3.86	0.44	2.26
25	0.45	2.36	0.67	0.86	0.67	0.92
50	0.71	1.32	0.79	0.84	0.79	0.85
100	0.73	2.36	0.82	1.35	0.83	1.26

Supplement Table 2. The CI value of CA with DTIC in different melanoma cells.

DTIC	CA (10 μM)		CA (20 μM)		CA (40 μM)	
C918	Fa	CI	Fa	CI	Fa	CI
1.56	0.19	1.06	0.3	1.24	0.47	1.52
3.13	0.23	1.14	0.41	1.04	0.55	1.33
6.25	0.31	1.18	0.57	0.81	0.67	1.06
12.5	0.51	0.92	0.63	0.87	0.73	1.01
25	0.6	1.11	0.73	0.87	0.78	1.04
50	0.73	1.16	0.84	0.78	0.85	0.99
100	0.84	1.17	0.9	0.82	0.93	0.75
200	0.92	1.08	0.93	1.02	0.93	1.18
DTIC	CA (10 μM)		CA (20 μM)		CA (40 μM)	
A875	Fa	CI	Fa	CI	Fa	CI
1.56	0.12	1.87	0.34	1.22	0.41	1.83
3.13	0.23	1.48	0.44	1.1	0.5	1.61
6.25	0.42	1.13	0.46	1.36	0.55	1.65
12.5	0.52	1.32	0.63	1.16	0.69	1.37
25	0.71	1.15	0.73	1.25	0.79	1.27
50	0.83	1.14	0.83	1.28	0.89	1.29
100	0.92	0.99	0.91	1.2	0.94	0.97
200	0.95	1.21	0.98	0.55	0.96	1.15
DTIC	CA (10 μM)		CA (20 μM)		CA (40 μM)	
A375	Fa	CI	Fa	CI	Fa	CI
1.56	0.115	1.64	0.38	1.22	0.54	1.37
3.13	0.28	1.82	0.44	1.26	0.6	1.28
6.25	0.58	0.75	0.58	1.03	0.71	1.03
12.5	0.63	0.96	0.68	0.97	0.78	0.93
25	0.67	1.36	0.82	0.68	0.89	0.6
50	0.75	1.54	0.88	0.64	0.9	0.71
100	0.89	0.84	0.94	0.45	0.95	0.5
200	0.91	1.19	0.98	0.21	0.97	0.43
DTIC	CA (10 μM)		CA (20 μM)		CA (40 μM)	
SK-MEL-1	Fa	CI	Fa	CI	Fa	CI
1.56	0.28	0.76	0.41	0.94	0.48	1.49
3.13	0.37	0.72	0.42	1.03	0.55	1.34
6.25	0.42	0.85	0.5	1.01	0.59	1.34
12.5	0.53	0.93	0.56	1.14	0.63	1.43
25	0.69	0.89	0.71	1.04	0.77	1.16
50	0.75	1.24	0.76	1.36	0.81	1.37
100	0.91	0.83	0.9	1.01	0.91	1.1
200	0.91	1.57	0.93	1.32	0.94	1.3

Part Two

In this section, we have provided all the original blots. Each experiment was repeated three times. We have marked the molecular weight markers on the Western blotting images in the final figures of the manuscript.

The molecular weight marker that we have used in the current study was purchased from Thermo Fisher Scientific (Cat. 26616/26619, Shanghai, China). We have used the Chemiluminescence imaging system (Cat. Tanon-5200, Shanghai, China). Image software was used for all sensitivity analyses, and we adopt the relative quantitative statistics and normalized the value.

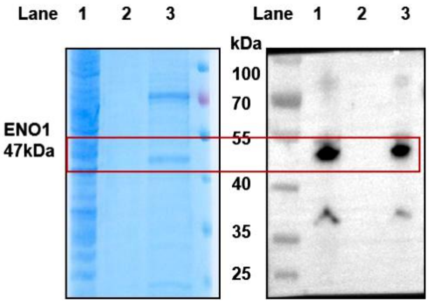

Figure S12. Original unedited pictures for the efficiency of ENO1 magnetic capture by SDS-PAGE and Western blotting (Figure 2B). Lane 1 shows the A375 lysate as loading control; Lane 2 shows the lysate captured only by the azide-modified MMs as a negative control; Lane 3 shows the lysate captured by AL-CA-MMs. This experiment is a qualitative analysis.

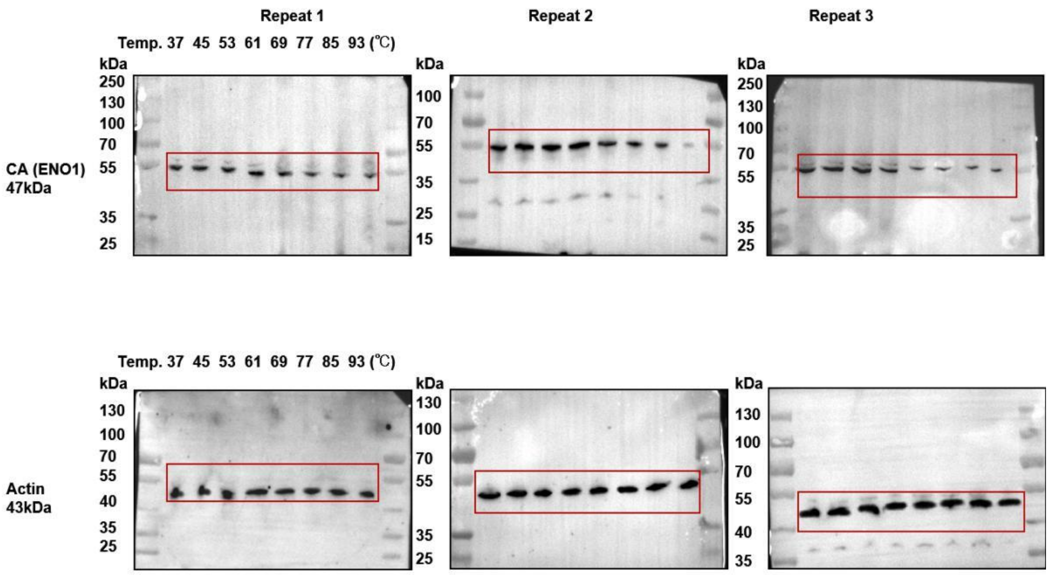

		Repeat 1		Repeat 2		Repeat 3						
CA 10 μM	Temp.(℃)	intden	Normalization to Actin (with same group)	Quadratic normalizatio n to NC (in ENO1 group)	intden	Normalizatio n to Actin (with same group)	Quadratic normalizatio n to NC (in ENO1 group)	intden	Normalizatio n to Actin (with same group)	Quadratic normalizatio n to NC (in ENO1 group)	The mean value of the three normalize	The standard deviation of the three normalized groups
	37	279126	1.055089359	1	272586	0.718146108	1	143767	0.571195296	1	1	0
	45	269119	1.087626345	1.030838134	241164	0.689919154	0.960694692	138048	0.587335827	1.028257464	1.0065968	0.039773296
	53	265720	1.039637856	0.985355266	222988	0.662593823	0.922644871	128684	0.540026522	0.94543237	0.9511442	0.031742982
	61	227955	0.844237129	0.800156994	189352	0.630200724	0.877538312	122575	0.425318186	0.7446108	0.8074354	0.06676198
	69	215392	0.91287524	0.865211304	111679	0.344537271	0.479759295	124486	0.406487553	0.711643733	0.6855381	0.194047521
	77	183889	0.828172148	0.784930813	107716	0.351470934	0.489414243	99428	0.393513993	0.688930731	0.6544253	0.150749731
	85	110392	0.494054359	0.468258309	106843	0.294921552	0.410670682	92610	0.419053566	0.733643237	0.5375241	0.172267605
	93	81145	0.400219974	0.379323297	90252	0.244093871	0.339894442	67889	0.277529546	0.485875055	0.4016976	0.075518487
Actin	Temp.(℃)	intden			intden			intden				
	37	264552			379569			251695				
	45	247437			349554			235041				
	53	255589			336538			238292				
	61	270013			300463			288196				
	69	235949			324142			306248				
	77	222042			306472			252667				
	85	223441			362276			220998				
	93	202751			369743			244619				

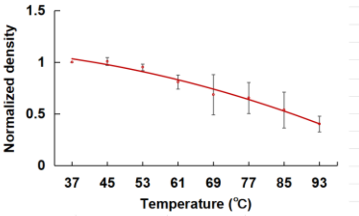

Figure S13. Original unedited bands for evaluating CA treatment (10 μM) decreased the thermal stability of ENO1 in cell lysates measured by the temperature-dependent CETSA in Figure 2C. Image software was used for sensitivity analysis, and the relative quantitative statistics and normalized the intensity value against 37ºC were adopted. Western blotting was performed 3 times. The "Repeat 2" was used in Figure 2C of the manuscript.

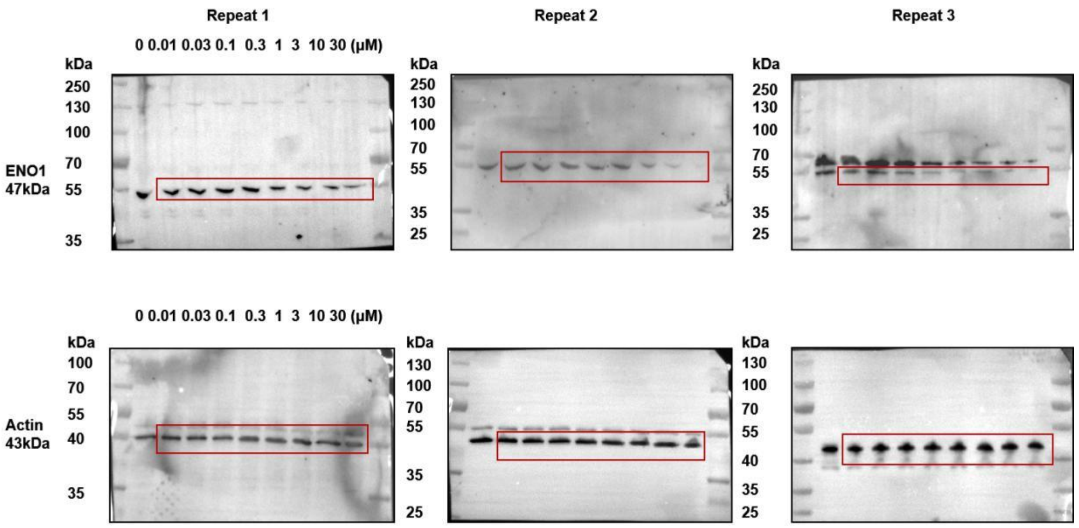

ENO1 (69°C)	Concentration (μM)	Repeat 1 intden	Normalization to Actin (with same group)	Quadratic normalization to NC (in ENO1 group)	Repeat 2 intden	Normalization to Actin (with same group)	Quadratic normalization to NC (in ENO1 group)	Repeat 3 intden	Normalization to Actin (with same group)	Quadratic normalization to NC (in ENO1 group)	The mean value of the three normalized	The standard deviation of the three normalized groups
	0	228391	1.324220743	1	166412	0.478250374	1	218467	1.022091745	1	1	0
	0.01	215502	1.051983618	0.794417111	123562	0.379159456	0.792805351	191638	0.998603483	0.977019419	0.854747294	0.105893833
	0.03	208719	1.030090513	0.777884291	141208	0.454243656	0.949803035	152930	0.70092537	0.685775395	0.804487574	0.13400914
	0.1	216355	1.148917754	0.867618001	121536	0.399642237	0.835633926	115549	0.62898871	0.615393592	0.772881839	0.137323182
	0.3	215021	1.139926946	0.860828492	126497	0.405536587	0.847958747	99748	0.537180647	0.525569891	0.74478571	0.189955492
	1	151356	0.73147817	0.552383863	100942	0.313062518	0.654599631	71056	0.441992249	0.432438919	0.546474138	0.111198198
	3	128884	0.609377733	0.460178363	92836	0.330612536	0.691295928	53264	0.344996438	0.337539599	0.496337963	0.179628847
	10	114453	0.630584618	0.476192977	65074	0.22358204	0.46749998	62619	0.391684546	0.383218579	0.442303846	0.051353614
	30	93367	0.528153637	0.398841085	45725	0.167543622	0.35032617	23678	0.156778875	0.153390218	0.300852491	0.129989483

Actin (69°C)	Concentration (μM)	Repeat 1 intden	Repeat 2 intden	Repeat 3 intden
	0	172472	347960	213745
	0.01	204853	325884	191906
	0.03	202622	310864	218183
	0.1	188312	304112	183706
	0.3	188627	311925	185688
	1	206918	322434	160763
	3	211501	280800	154390
	10	181503	291052	159871
	30	176780	272914	151028

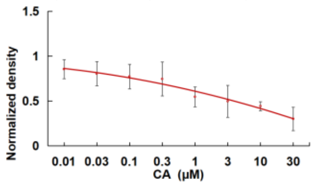

Figure S14. Original unedited bands for evaluating CA treatment decreased the thermal stability of ENO1 in cell lysates measured by the concentration-dependent CETSA at 69 °C in Figure 2D. In order to unify the number of strips in Figure 2C, the first strip (0 μM) was cut off in this figure as it corresponds to a negative control. Image software was used for sensitivity analysis, and the relative quantitative statistics and normalized the intensity value against CA unadded control group (0 μM) were adopted. Western blotting was performed 3 times. The "Repeat 1" was used in Figure 2D of the manuscript.

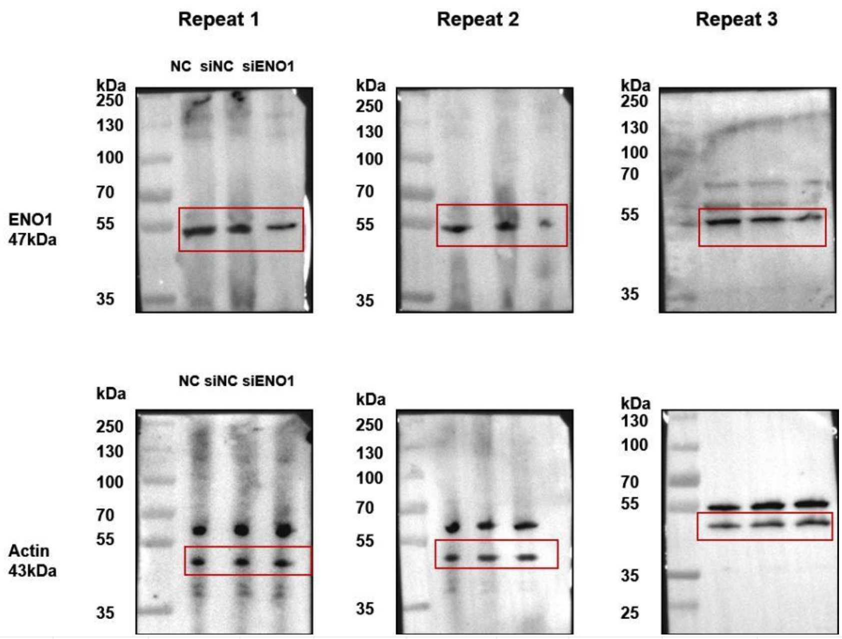

		Repeat 1			Repeat 2			Repeat 3				
ENO1	Groups	intden	Normalization to Actin (with same group)	Quadratic normalization to NC (in ENO1 group)	intden	Normalization to Actin (with same group)	Quadratic normalization to NC (in ENO1 group)	intden	Normalization to Actin (with same group)	Quadratic normalization to NC (in ENO1 group)	The mean value of the three normalized groups	The standard deviation of the three normalized groups
	NC	239376	2.393042087	1	202009	1.448352751	1	181638	1.026000508	1	1	0
	siNC	233876	2.188723036	0.914619533	215948	1.581538416	1.091956649	163422	0.795167357	0.775016533	0.927197571	0.158843994
	siENO1	132559	1.257043422	0.52529098	80456	0.541648995	0.37397588	119887	0.498451266	0.485819706	0.461695522	0.078489152
Actin	NC	100030			139475			177035				
	siNC	106855			136543			205519				
	siENO1	105453			148539			240519				

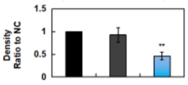

Figure S15. Original unedited bands for evaluating CA inhibited ENO1 function in Figure 4D. A375 cells were transfected with siENO1 for 48 h, and the expression of ENO1 in cells was assayed by Western blotting 3 times. Image software was used for sensitivity analysis, and the relative quantitative statistics and normalized the density value of the control group were adopted. The "Repeat 3" was used in Figure 4D of the manuscript.
